# Advancing the performance characteristics of rubber asphalt mixtures through the integration of Basic Oxygen Furnace (BOF) slag: A focus on static and dynamic mechanical enhancements

**DOI:** 10.1371/journal.pone.0310499

**Published:** 2024-09-18

**Authors:** Zhongnan Tian, Peng Liang, Bochao Zhai, Yue Zhou

**Affiliations:** 1 Hebei Provincial Communications Planning, Design and Research Institute Co., Ltd, Shijiazhuang, Hebei, China; 2 Chang’an University, Xi’an, Shaanxi, China; 3 Shijiazhuang Tiedao University, Shaanxi, China; 4 Hebei Provincial Department of Transportation, Shijiazhuang, Hebei, China; 5 Hebei Xiong’an Transportation Investment Co., Ltd, Xiong’an, Hebei, China; 6 Hebei Jiaotong Vocational and Technical College, Shijiazhuang, Hebei, China; Shandong University of Technology, CHINA

## Abstract

To investigate the advantageous effects of incorporating industrial solid waste basic oxygen furnace (BOF) slag on the mechanical characteristics of warm-mixed rubber asphalt (WMRA) and hot-mixed rubber asphalt (HMRA) mixture, varying proportions of BOF slag were substituted for limestone coarse aggregates (0%, 25%, 50%, and 75%). Additionally, a 1.5% dosage of Sasobit warm-mixed modifier was introduced to prepare the rubber asphalt. Subsequent to preparation, both static mechanical tests (including Marshall and indirect tensile tests) and dynamic mechanical tests (including dynamic creep and elastic modulus tests) were conducted to evaluate the influence of BOF slag on the mechanical behavior of WMRA and HMRA mixtures across different substitution levels. Following testing, a two-way analysis of variance (ANOVA) was employed to dissect the impact of BOF slag content and Sasobit warm-mixed modifier on the static and dynamic mechanical properties of the rubber asphalt mixtures. The findings reveal that BOF slag exhibits commendable engineering aggregate properties, enabling substantial substitution of coarse aggregates in both HMRA and WMRA mixtures. As the proportion of BOF slag increases, it enhances the resistance of asphalt mixtures to permanent deformation and cracking under static and dynamic loading conditions, while broadening the range of elastic deformation for both WMRA and HMRA mixtures subjected to repeated loading. Moreover, a synergistic enhancement in the resistance of rubber asphalt mixtures to dynamic load-induced deformation is observed when employing both BOF slag and Sasobit warm-mixed modifier. The findings offer valuable insights for enhancing the performance of WMRA and HMRA mixtures, as well as broadening the utilization of BOF slag and waste rubber.

## 1. Introduction

The “Carbon Neutrality and Carbon Peaking” strategies and the Fourteenth Five-Year Plan, proposed by the Chinese government, have placed stringent demands on ecological health. The means of hilltop deforestation and aggregate collection for engineering materials have been strictly restricted, resulting in a significant decrease in the supply of engineering aggregates. Asphalt mixtures require a large amount of coarse and fine aggregates, especially coarse aggregates, with every 100 m^3^ of asphalt pavement consuming 723.2 m^3^ of coarse aggregates. Steel slag, as a by-product of steel production, exceeds 100 million tons annually, with basic oxygen furnace (BOF) slag accounting for about 60–80% of the steel slag production [1, 2]. BOF slag is generated during the steelmaking process using scrap steel in a BOF slag and contains over 20% iron. It possesses advantages such as low crush value, abrasion resistance, high density, and good angularity, indicating significant potential for replacing engineering aggregates. Additionally, warm-mixed asphalt (WMRA) technology can reduce asphalt mixture mixing and construction temperatures by 40–60°C, substantially decreasing fuel consumption and carbon emissions [3–5]. Adding BOF slag as coarse aggregate in WMRA not only enhances the road performance of WMRA but also alleviates aggregate supply pressures, reduces project costs, and enhances environmental benefits [5–7].

Due to its high strength and angularity, BOF slag enhances the high-temperature deformation resistance of asphalt mixtures [8]. Masoudi found a significant improvement in the rutting resistance of asphalt mixtures subjected to moisture-temperature coupling when 50% steel slag was added to the mixture [9]. Hesami analyzed the water stability of asphalt mixtures containing BOF slag using parameters such as Marshall stability ratio and resilient modulus ratio, concluding that the addition of BOF slag enhances the resistance of asphalt mixtures to moisture-induced damage [10]. Liu Y evaluated the long-term aging performance of steel slag warm-mixed asphalt mixtures and observed a reduction in aging length and noticeable performance improvement compared to blank samples [11]. Shen believes that the porous structure of BOF slag can effectively improve the adhesion between BOF slag and rubber asphalt, accompanied by this weak chemical reaction [12]. The unique characteristics of BOF slag, including high strength, good angularity, and excellent adhesion to asphalt, significantly enhance the rutting resistance of warm-mixed asphalt mixtures, thereby expanding their application scope. However, as road vehicle loads primarily consist of dynamic loads, the significant difference in stiffness and modulus between BOF slag and limestone coarse aggregates results in noticeable differences in the viscoelastic range and deformation of warm-mixed asphalt mixtures containing BOF slag under dynamic and static loading [13–15]. This discrepancy impacts the long-term performance and service life of asphalt mixtures with added BOF slag under actual loading conditions. At present, the research on WMRA and HMRA mixture mixed with steel slag is limited.

Therefore, aiming to investigate the advantageous effects of BOF slag on the static and dynamic mechanical characteristics of WMRA and HMRA mixture, this study employs BOF slag and limestone as coarse aggregates, substituting 0%, 25%, 50%, and 75% of limestone coarse aggregates, supplemented with 1.5% Sasobit warm-mixed modifier and 25% rubber modifier by weight of base asphalt. And the superpave gyratory compactor (SGC) is used to form WMRA and HMRA mixtures. Subsequently, static mechanical tests (Marshall test and indirect tensile test) and dynamic mechanical tests (dynamic creep test and resilient modulus test) are conducted to investigate the influence of different proportions of BOF slag on the static and dynamic mechanical properties of WMRA and HMRA mixtures. Furthermore, a two-way analysis of variance is employed to analyze the significant effects of BOF slag and Sasobit warm-mixed modifier on the static and dynamic mechanical properties of rubber asphalt mixtures The findings offer valuable insights for enhancing the performance of WMRA and HMRA mixtures, as well as broadening the utilization of BOF slag and waste rubber.

## 2. Materials and measurements

### 2.1. Materials

**Fig 1:**
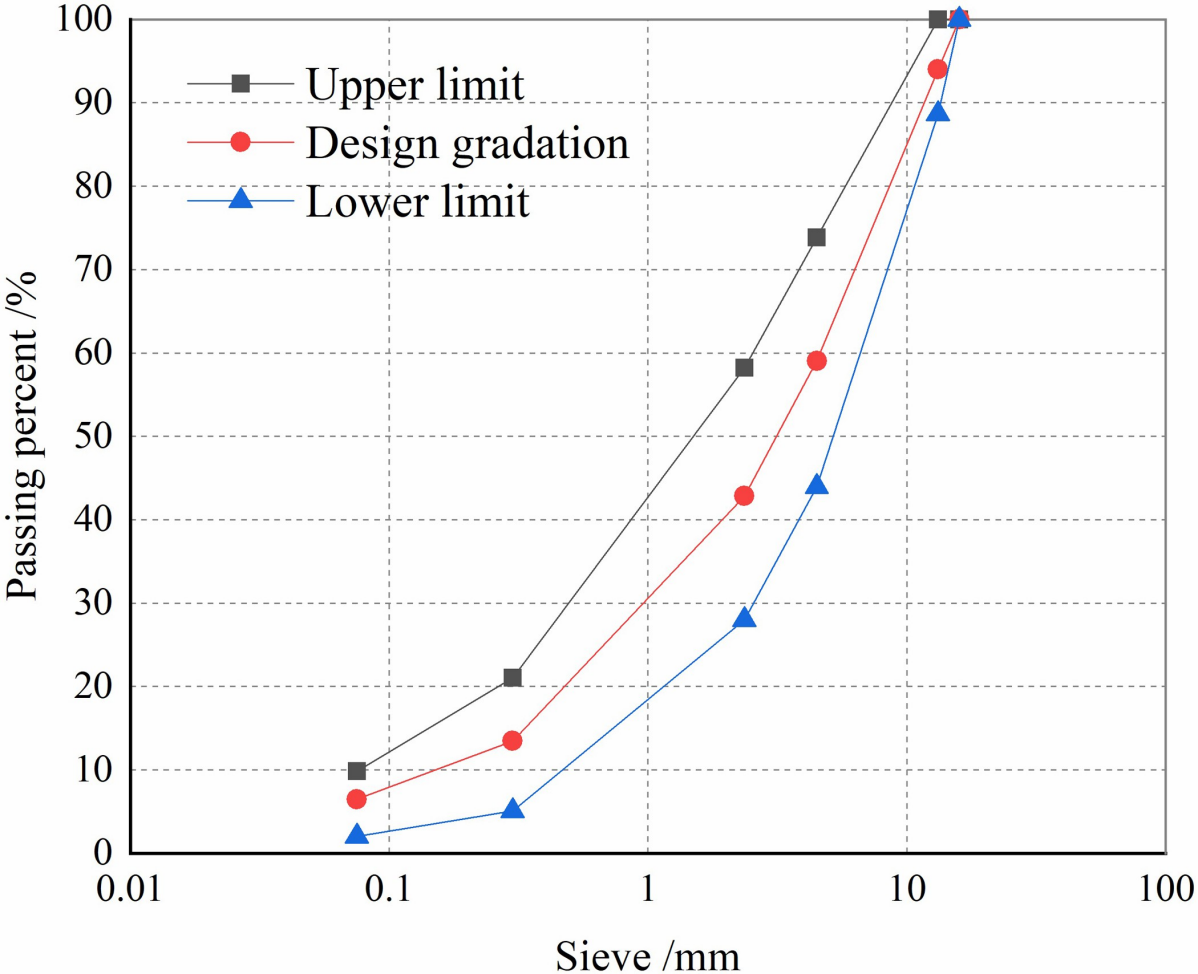


**Fig 2:**
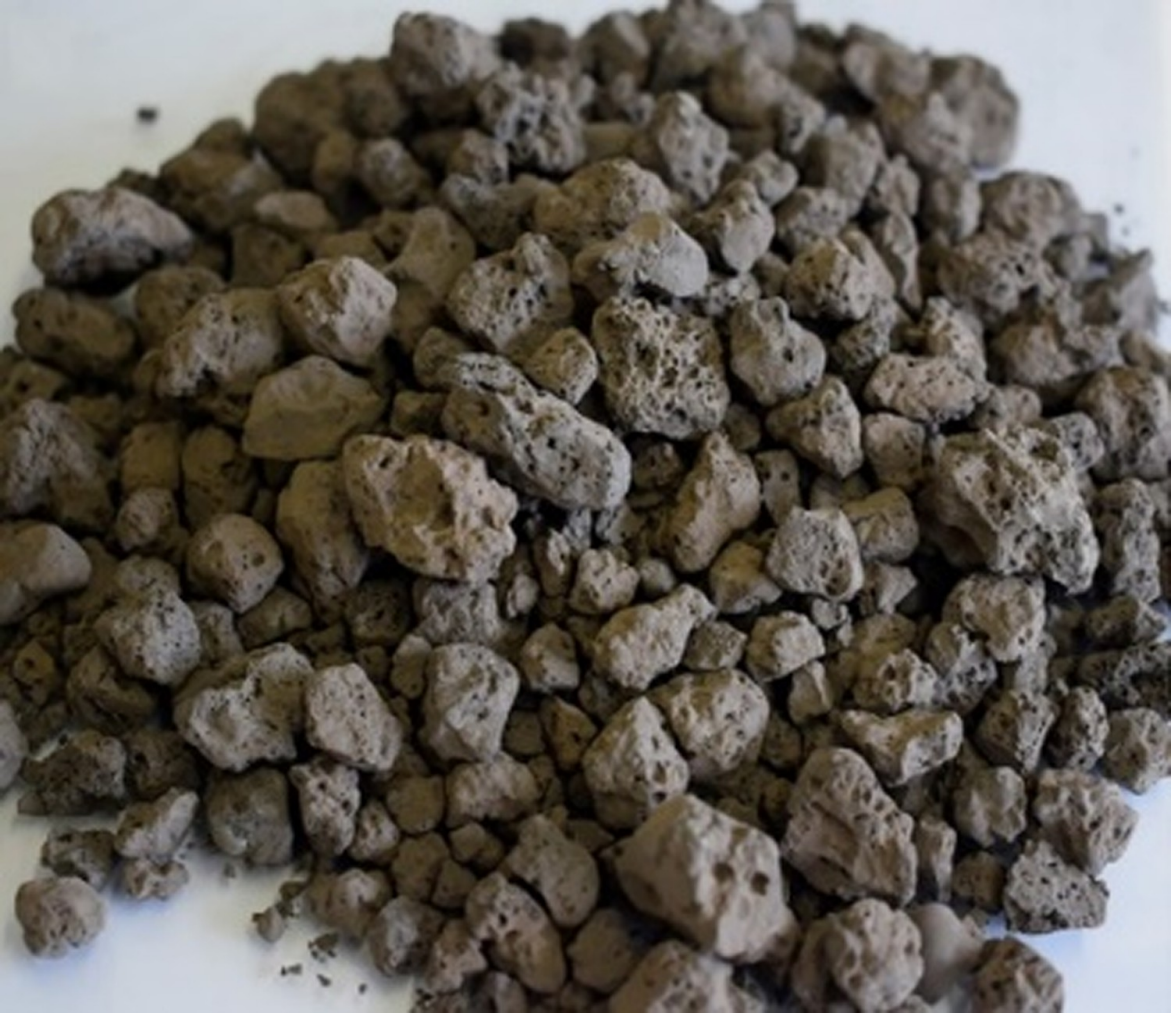


The basic oxygen furnace (BOF) slag used in this study is produced in Tangshan. The grading curves for BOF slag and limestone aggregates are presented in Fig 1. According to the JTG E42-2005 standard, the physical properties of BOF slag and limestone aggregates are shown in Table 1. BOF slag aggregates exhibit excellent mechanical strength, abrasion resistance, and angularity compared to limestone aggregates. Table 2 presents the chemical composition of BOF slag and limestone aggregates as determined by X-ray fluorescence (XRF) analysis. BOF slag aggregates are primarily composed of CaO, Fe_2_O_3_, and SiO_2_, with a high Fe_2_O_3_ content of up to 25.13%, contributing to their high strength and abrasion resistance. Fig 2 illustrates the surface structure of coarse aggregates from BOF slag. BOF slag aggregates exhibit larger surface pores, roughness, and compactness, while limestone aggregates have a relatively smooth and even surface. The asphalt chosen for this study is rubber asphalt with 25% rubber modifier added, and its performance indicators are listed in Table 3. The warm-mixed modifier used is Sasobit, which is pale yellow in color and granulated, with a melting point of 105°C, and is added at a dosage of 1.5% of the base asphalt mass.

**Table 1:** Physical properties of BOF slag and limestone coarse aggregate.

Test item	Limestone	BOF slag	Requirement
Bulk density /g·cm^-3^	2.718	3.355	-
Apparent density/g·cm^-3^	2.635	3.456	≧2.60
Water absorption rate/%	1.71	1.39	≤2.0
Crushing value/%	20.9	9.7	≤ 26
Los Angles abrasion/%	24.2	13.3	≤ 28
Elongated particles/%	9.4	2.1	≤ 18

**Table 2:** Chemical composition of limestone and BOF slag in XRF test (%).

Aggregate	SiO_2_	TiO_2_	CaO	Fe_2_O_3_	MgO	Al_2_O_3_	P_2_O_5_	K_2_O	MnO	Na_2_O	SO_3_
Limestone	12.11	0.09	36.49	1.00	8,83	1.56	0.08	0.47	0.03	0.21	0.01
BOF slag	21.23	0.71	27.85	25.13	5.69	5.52	0.47	0.69	0.39	0.82	0.27

**Table 3:** Performance indicators of rubber asphalt used.

Test items	Value
Rotational viscosity/(180°C, Pa·s)	2.7
Penetration/(15°C,0.1mm)	66
Softening point /°C	71
Ductility /cm	17
Separation softening point difference /°C	1.8
Elastic recovery /%	83

### 2.2 Preparation of mixtures

As shown in Table 4, HMRA and WMRA mixtures were prepared by substituting different proportions of limestone coarse aggregates with BOF slag. The substitution rates for BOF slag were 0%, 25%, 50%, and 75%. Sasobit warm-mixed modifier, at a dosage of 1.5% of the asphalt mass, was added to the WMRA mixtures to reduce the mixing and compaction temperatures of the asphalt mixtures. Rubber modifier, at a dosage of 25% of the base asphalt mass, was added to the WMRA mixtures to improve the performance of the asphalt mixture. The mixing temperatures for WMRA and HMRA mixtures were set at 120°C and 160°C, respectively, while the compaction temperatures were determined to be 110°C and 150°C, respectively.

**Table 4:** Experiments design of WMRA and HMRA mixtures.

Abbreviation	Rubber content (%)	Sasobit content (%)	Coarse BOF slag content (%)
H0	25	0	0
H25	25	0	25
H50	25	0	50
H75	25	0	75
W0	25	1.5	0
W25	25	1.5	25
W50	25	1.5	50
W75	25	1.5	75

### 2.3 Experimental program

#### 2.3.1 Marshall test

Marshall testing was employed to measure the volume parameters of each asphalt mixture and determine the optimal asphalt content. Subsequently, cylindrical Marshall specimens at the optimal asphalt content were immersed in a 60°C water bath, and the Marshall testing apparatus was utilized to apply load for measuring their stability and flow values. The Marshall modulus, calculated as the ratio of stability to flow values, serves as a metric for asphalt mixture stiffness. It provides insight into the resistance to permanent deformation of asphalt mixtures at 60°C, with a higher Marshall modulus indicating greater stiffness and resistance to permanent deformation.

#### 2.3.2 Indirect tensile strength test

Indirect tensile strength (ITS) is a strength indicator used to evaluate the crack resistance of asphalt mixtures. In this study, the effect of BOF slag content on the crack resistance of WMRA and HMRA mixtures was analyzed through indirect tensile strength testing. The experiments involved cylindrical specimens formed by rotational compaction, tested at a temperature of 25°C, and subjected to a radial load at a rate of 50 mm/min until failure. The tensile stress generated in the direction perpendicular to the loading surface is calculated according to Eq (1).


$$ITS=\frac{2P_{\max}}{\pi td}$$ (1)

Where, *ITS* represents the indirect tensile strength (MPa), *P*_*max*_ is the maximum load at failure (kN), and *t* and *d* denote the height and diameter of the cylindrical specimen (mm).

#### 2.3.3 Dynamic creep test

**Fig 3:**
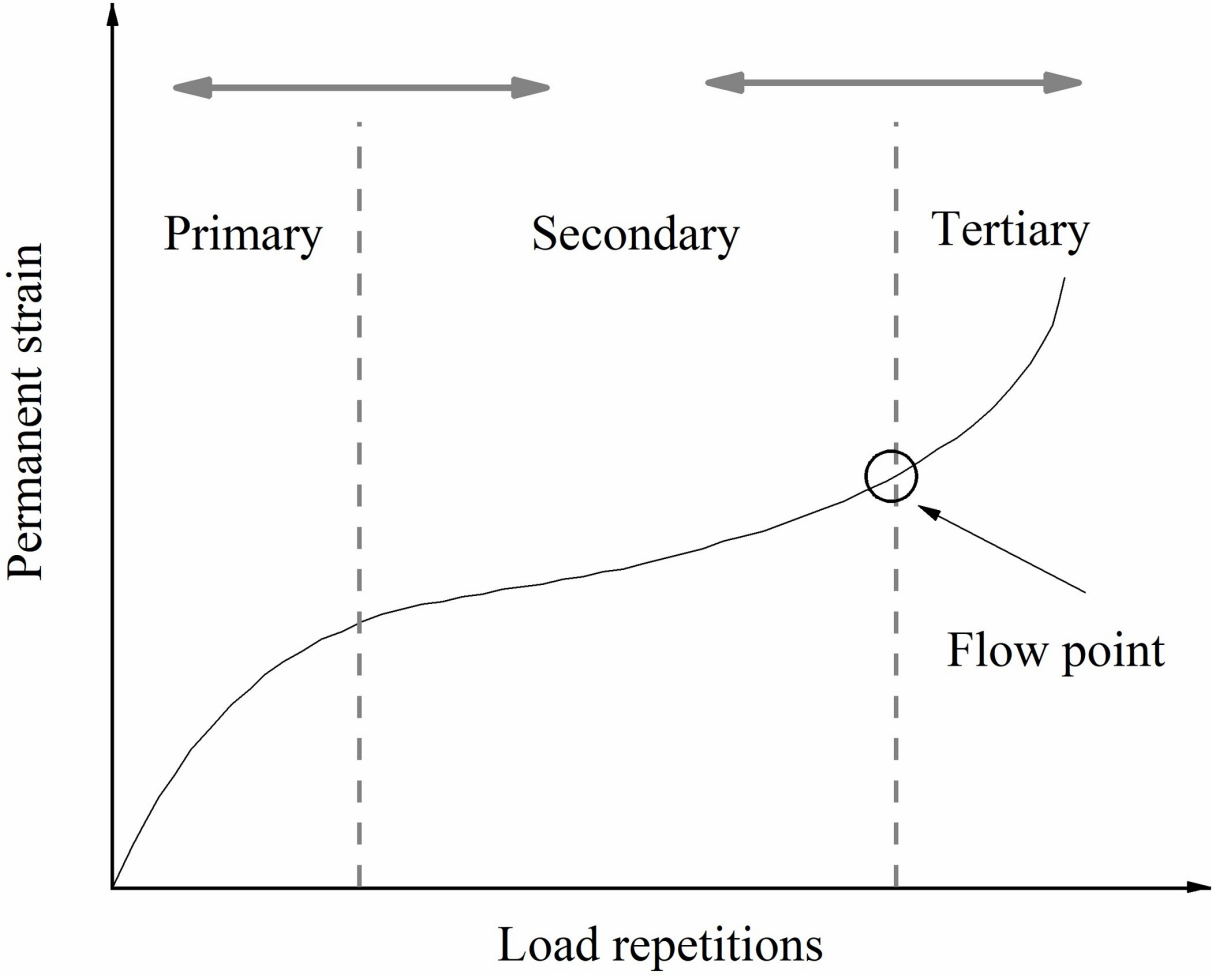


The dynamic creep test is a method used to assess the shear deformation resistance of asphalt mixtures. In this study, the effect of BOF slag content on the dynamic resistance to permanent deformation of WMRA and HMRA mixtures was analyzed through dynamic creep testing. This test involves applying dynamic loads to cylindrical specimens and measuring their deformation to obtain a dynamic creep curve under load, allowing for the study of the mixture’s resistance to permanent deformation [16, 17]. Fig 3 illustrates the permanent deformation curve of asphalt mixtures under cyclic dynamic loading. As shown in Fig 3, asphalt mixtures undergo three stages under load, with primary deformation occurring in the first and last stages. In the first stage, strain is induced by volume compression of the mixture, while in the third stage, the main strain is caused by shear deformation resulting in the failure of asphalt mixture strength. The starting point of the third stage is called as the flow number, representing the number of cycles at which shear deformation failure begins in the asphalt mixture. It is used to evaluate the dynamic resistance to permanent deformation performance of the mixture.

**Fig 4:**
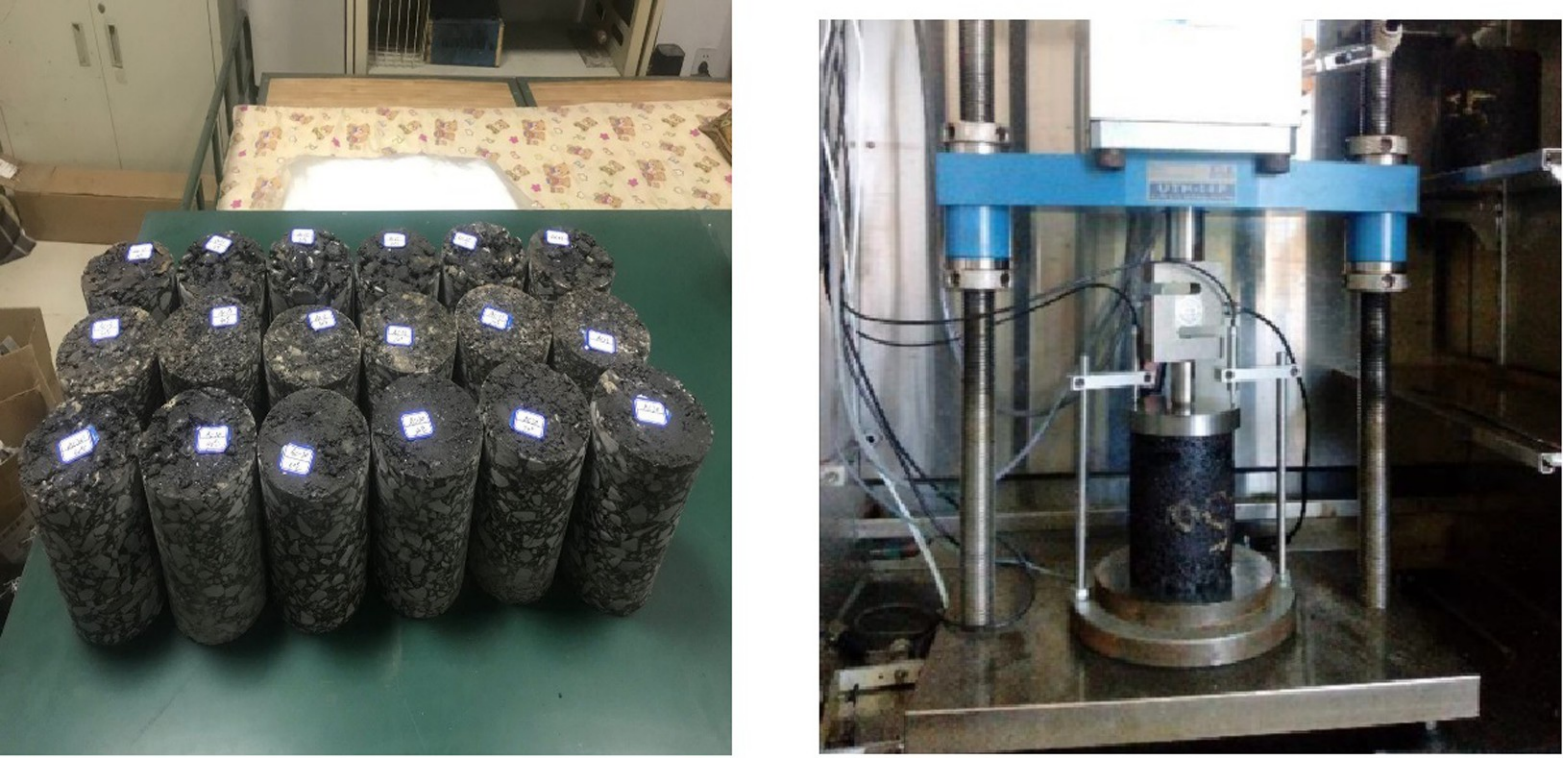


The dynamic creep tests were conducted using the UTM-50 testing system following the specifications outlined in NCHRP 9–29. Cylindrical specimens with a diameter of 100 mm and a height of 150 mm were fabricated using a superpave gyratory compactor (SGC) at the optimum asphalt content, with a controlled void ratio of 4%. The tests were performed at temperatures of 40°C and 50°C, in accordance with the procedures detailed in NCHRP 9–29. The flow numbers for different specimens were obtained by plotting the cumulative permanent deformation against the number of load cycles. The dynamic creep test specimens and equipment are depicted in Fig 4.

#### 2.3.4 Elastic modulus test

Asphalt mixtures exhibit viscoelastic behavior, where within a certain load range, elastic deformation is reversible while viscous deformation leads to permanent, irrecoverable changes. Under relatively small and repetitive loads, asphalt mixtures experience elastic deformation, which can almost entirely recover [10]. The elastic modulus, defined as the ratio of repetitive axial stress to recoverable strain, reflects the viscoelastic mechanical properties of asphalt mixtures. The effect of BOF slag content on the elastic modulus of WMRA and HMRA mixtures was analyzed using elastic modulus tests.

The elastic modulus tests were conducted following the Standard Test Method for Indirect Tensile Strength of Asphalt Mixtures (ASTM D 4123–82). Cylindrical specimens were fabricated using a gyratory compactor with a void ratio of 4%. According to ASTM D 4123–82, fatigue and rutting damage analyses were performed on asphalt mixtures containing BOF slag at 25°C and 40°C. Each specimen underwent 200 cycles of preloading followed by five cycles of sinusoidal loading radially, with deformations measured perpendicular to the loading direction. In this experiment, the loading and dwell times were 0.1s and 0.9s, respectively. The elastic modulus of the asphalt mixture was determined by Eq (2).


$$M_{r}=\frac{P(\mu +0.27)}{t\delta_{h}}$$ (2)

In Eq (2), *Mr* represents the elastic modulus (MPa); *t* stands for the length (mm); *μ* denotes the Poisson’s ratio, which is taken as 0.35; *P* signifies the maximum dynamic load applied to the specimen (N); *δ*_*h*_ represents the corresponding horizontal elastic deformation of the specimen under the maximum load (mm).

## 3 Results and discussion

### 3.1 Marshall test results

**Fig 5:**
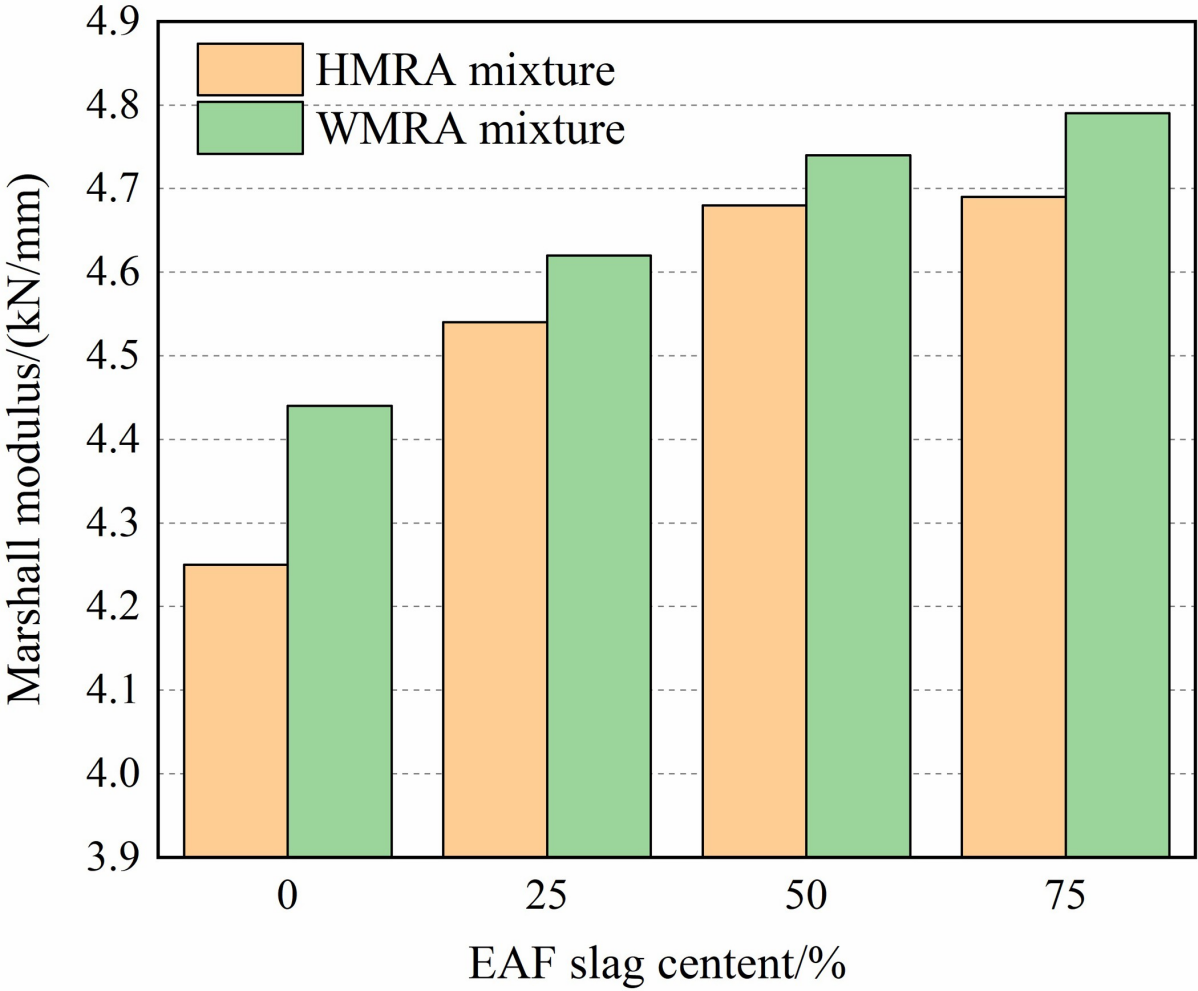


Table 5 presents the Marshall test results for HMRA and WMRA under different proportions of BOF slag. It is evident that the optimal asphalt content increases with the addition of BOF slag due to its high porosity and asphalt absorption rate. Moreover, the Marshall stability of both WMRA and HMRA mixtures gradually increases with the rising content of BOF slag, attributed to its sharp edges, significant surface roughness, and internal friction angle. Compared to W0 and H0, the Marshall stability of W75 and H75 respectively improves by 27% and 14%. The Marshall modulus, obtained by dividing stability by flow value, is depicted in Fig 5. It is observed that the Marshall modulus of both WMRA and HMRA mixtures increases with the increment of BOF slag content, indicating that augmenting the content of BOF slag enhances the stiffness of WMRA and HMRA mixtures, thereby bolstering their resistance to permanent deformation. Additionally, the incorporation of Sasobit warm-mixed modifier not only reduces the mixing and compaction temperatures of WMRA mixtures but also enhances the Marshall stability and modulus at 60°C, thereby reinforcing the resistance to permanent deformation of rubber asphalt mixtures.

**Table 5:** Marshall test results of HMRA and WMRA mixtures.

Mixture type	Asphalt dosage/%	Marshall stability/kN	Flow value/mm
H0	5.80	10.7	2.52
H25	6.00	11.8	2.60
H50	6.30	13.1	2.80
H75	6.50	13.6	2.90
W0	5.75	12.0	2.70
W25	6.00	12.7	2.75
W50	6.20	13.5	2.85
W75	6.40	13.7	2.86

### 3.2 Indirect tensile test results

**Fig 6:**
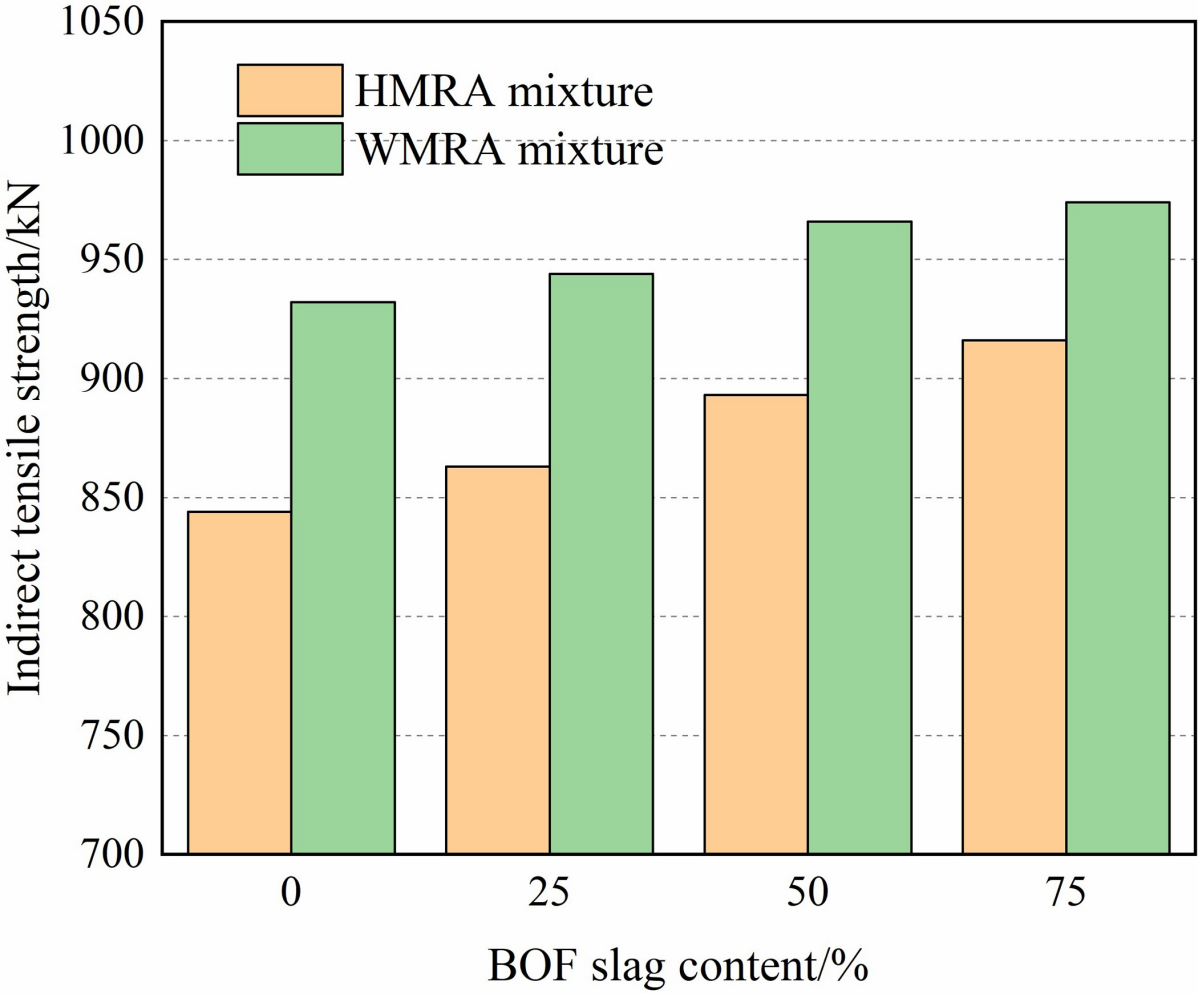


Fig 6 illustrates the results of the indirect tensile strength for HMRA and WMRA mixtures with varying proportions of BOF slag. The results indicate a gradual increase in indirect tensile strength with the addition of BOF slag. Compared to H0 and W0, the indirect tensile strength of H75 and W75 respectively increased by 9.6% and 5.1%. Since the tensile strength of rubber asphalt mixtures depends on the adhesion between rubber asphalt and aggregates, the excellent adhesion between asphalt and BOF slag reduces the risk of tensile failure between rubber asphalt and aggregates, consistent with the phenomenon observed by Shen [12] and Shu [18]. Moreover, the rough and porous surface of BOF slag increases the contact area between rubber asphalt and coarse BOF aggregates. Therefore, the crack resistance of WMRA and HMRA increases with the higher usage of coarse BOF slag aggregates.

Additionally, the crack resistance of WMRA mixtures with Sasobit warm-mixed modifier surpasses that of HMRA mixtures. This is attributed to the compatibility of Sasobit modifier, which saturates with rubber asphalt at room temperature, and its adsorption crystallization on the porous surface structure of BOF slag. This enhances the adhesion between rubber asphalt and BOF slag surface, thereby improving the crack resistance of WMRA mixtures.

### 3.3 Dynamic creep test results

**Fig 7:**
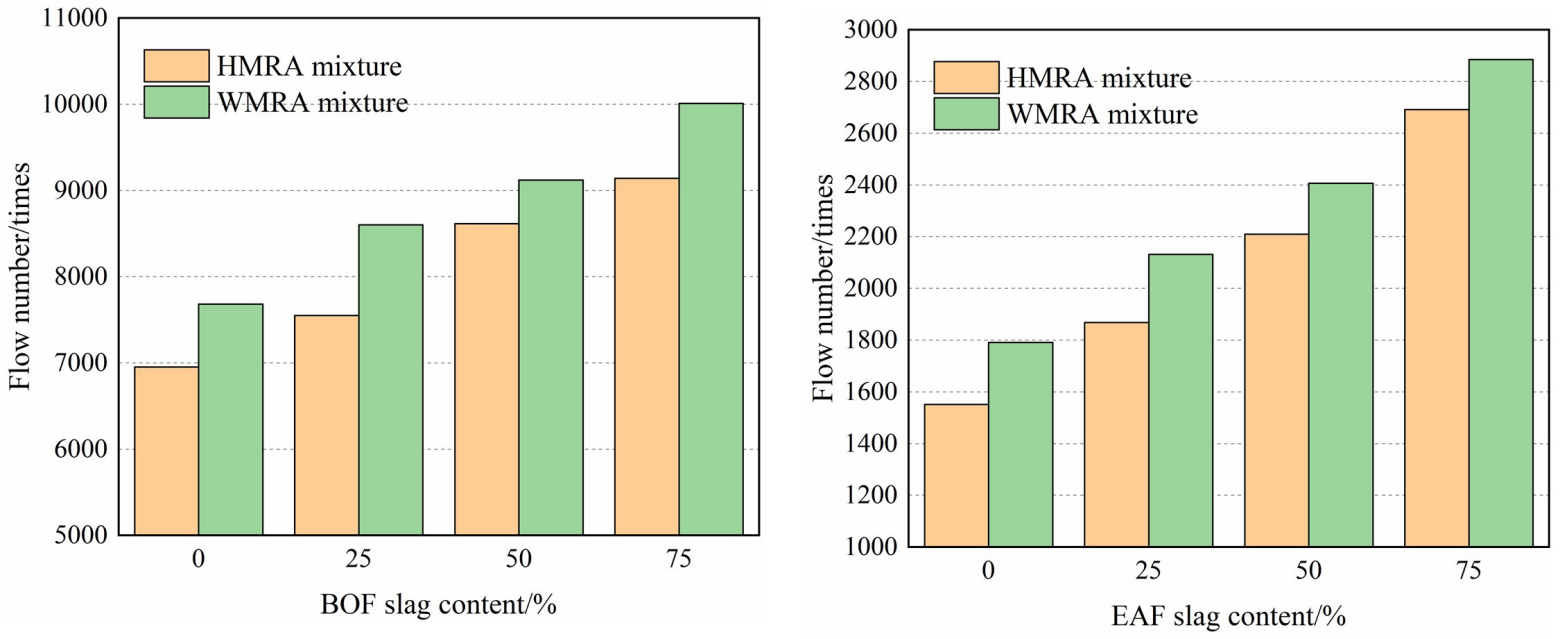


Dynamic creep tests is conducted at elevated temperatures to assess the resistance to permanent deformation of asphalt mixtures containing BOF slag. The resistance ability to high-temperature shear deformation of asphalt mixtures is characterized by obtaining the flow numbers until initial instability failure of the mixture under high-temperature conditions. Fig 7 presents the results of flow numbers for HMRA and WMRA mixtures with varying proportions of BOF slag at 40°C and 50°C. It is evident that compared to H0 and W0, the flow numbers of H75 and W75 increased by 31.5% and 30.3% at 40°C, and by 73.5% and 61.1% at 50°C, respectively. This indicates a substantial increase in flow numbers of HMRA and WMRA mixtures with the addition of BOF slag, primarily attributed to the sharp edges and internal friction angle of BOF slag.

Under the cyclic loading of dynamic creep tests, rubber asphalt binders gradually exhibit creep behavior under repeated stress, while the friction and interlocking action of aggregates significantly resist permanent deformation creep. Particularly, at high BOF slag contents, the internal friction angle of the mixture increases, thereby enhancing the resistance of HMRA and WMRA to permanent deformation under repeated loading.

Furthermore, the addition of 1.5% Sasobit warm-mixed modifier increased the flow numbers of WMRA mixtures. Under the same BOF slag content and temperature, WMRA mixtures with Sasobit modifier exhibited a 5.8% to 15.4% increase in flow numbers compared to HMRA mixtures. This indicates that WMRA mixtures with Sasobit modifier possess superior high-temperature resistance to deformation, primarily due to the modifying effect of Sasobit warm-mixed modifier on the high-temperature performance of rubber asphalt.

Sasobit warm-mixed modifier is a wax-based modifier that can exhibit a viscosity-reducing effect during the mixing and compaction stages of rubber asphalt mixtures when the temperature exceeds its melting point, by being compatible with asphalt saturates. When the temperature drops below its melting point, it absorbs some asphalt saturates and precipitates crystallization, increasing the viscosity of rubber asphalt within the working temperature range. Additionally, the interaction between Sasobit wax crystals and the high-calcium BOF slag aggregates is stronger, allowing them to penetrate and adsorb into the capillary pores on the surface of BOF slag, which is consistent with Cheng’s research results [19]. This enhances the adhesion between Sasobit-modified rubber asphalt and BOF slag, thereby improving the high-temperature resistance to permanent deformation of WMRA mixtures.

### 3.4 Elastic modulus test results

**Fig 8:**
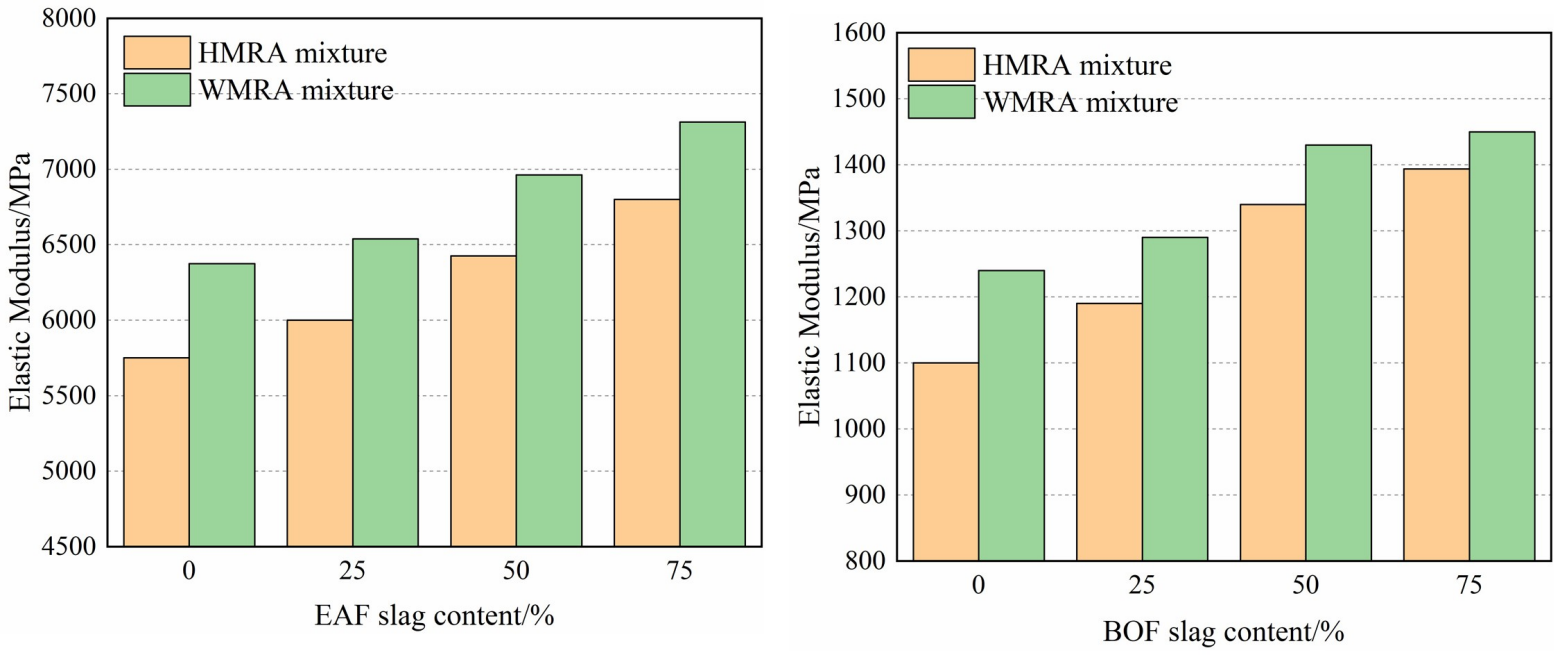


The distinctive surface structure and dosage of BOF slag aggregates positively impact the modulus of elasticity in both HMRA and WMRA compositions. Fig 8 illustrates the experimental findings regarding the modulus of elasticity variation in HMRA and WMRA mixtures with varying levels of BOF slag content, highlighting a significant positive correlation between increased coarse BOF slag aggregate dosage and enhanced modulus of elasticity in these mixtures. The modulus of elasticity at 25°C predominantly characterizes fatigue damage within the mixture, while at 40°C, it primarily characterizes rutting damage. A comparison between samples H75 and W75 with control samples H0 and W0 revealed an increase in modulus of elasticity at 25°C by 18.2% and 14.7%, respectively, and at 40°C by 26.7% and 16.9%, respectively. This indicates that the inclusion of coarse BOF slag aggregates improves medium-temperature fatigue resistance and high-temperature rutting deformation resistance in rubber asphalt mixtures.

Furthermore, the incorporation of Sasobit warm-mixed modifier resulted in increased modulus of elasticity at both 25°C and 40°C for WMRA mixtures compared to HMRA mixtures. In comparison to samples without BOF slag (H0 and W0), the greatest increase in modulus of elasticity at 25°C and 40°C was observed in W0 samples, reaching 10.8% and 12.7%, respectively. As the dosage of BOF slag increased, the increment in modulus of elasticity of WMRA mixtures relative to HMRA mixtures gradually decreased. This suggests a synergistic enhancement effect of Sasobit warm mix modifier and BOF slag on the fatigue resistance at medium-temperature and rutting resistance of rubber asphalt mixtures at high-temperature, thereby expanding the elastic deformation range of both WMRA and HMRA mixtures while reducing permanent deformation in these mixtures [20, 21].

Dynamic loading tests (including dynamic creep and modulus of elasticity tests) and static loading tests (such as Marshall and indirect tensile tests) revealed that BOF slag and Sasobit warm-mixed modifier significantly enhance the resistance of rubber asphalt mixtures to dynamic deformation. Furthermore, their impact on improving high-temperature mechanical performance surpasses their influence on enhancing low-temperature cracking resistance. The synergistic effect of BOF slag and Sasobit warm-mixed modifier in fortifying the resistance of rubber asphalt mixtures to both dynamic and static deformation showcases a collaborative reinforcement, thus enhancing the dynamic and static mechanical properties of rubber asphalt mixtures simultaneously.

## 4 Analysis of variance (ANOVA)

To further elucidate the synergistic impact of BOF slag aggregates and Sasobit warm-mixed modifier on the dynamic and static mechanical properties of rubber asphalt mixtures, a two-way analysis of variance (ANOVA) was conducted using SPSS statistical software, assessing the results of dynamic and static mechanical tests of rubber asphalt mixtures at a 95% confidence level [22, 23]. The results are summarized in Table 6.

**Table 6:** Results of two-way ANOVA.

Indicators	Source of variance	Sum of squares	Degrees of freedom	Mean square	F-value	P-value	Significance*
Marshall modulus (60°C)	Sasobit	0.124	1	0.124	8.967	0.010	**
	BOF	0.393	3	0.131	9.476	0.001	***
	Sasobit*BOF	0.009	3	0.003	0.211	0.886	
	Error	0.221	16	0.014			
	Total	0.746	23				
Indirect tensile strength (25°C)	Sasobit	11250	1	11250	20.925	0.000	***
	BOF	8925	3	2975	5.533	0.011	*
	Sasobit*BOF	449	3	149	0.278	0.840	
	Error	8602	16	537			
	Total	29226	23				
Flow number (40°C)	Sasobit	653352	1	653352	8.263	0.013	*
	BOF	2547432	3	849144	10.739	0.000	***
	Sasobit*BOF	834975	3	278325	3.520	0.045	*
	Error	1265082	16	79067			
	Total	5300841	23				
Flow number (50°C)	Sasobit	84402	1	84402	3.285	0.093	
	BOF	601611	3	200537	7.805	0.003	*
	Sasobit*BOF	2787	3	929	0.036	0.990	
	Error	411062	16	25691			
	Total	1099862	23				
Elastic modulus (25°C)	Sasobit	97903	1	97903	13.923	0.002	**
	BOF	188859	3	62953	8.953	0.001	***
	Sasobit*BOF	46584	3	15528	2.208	0.135	
	Error	112501	16	7031			
	Total	445847	23				
Elastic modulus (40°C)	Sasobit	4656	1	4656	7.841	0.015	*
	BOF	21268	3	7089	11.938	0.000	***
	Sasobit*BOF	6448	3	2149	3.619	0.042	*
	Error	9501	16	593.813			
	Total	41873	23				

* Significance level: 0.01<P ≤ 0.05 is marked as "*", 0.001<P ≤ 0.01 is marked as "* *", and P ≤ 0.001 is marked as "***".

The analysis reveals distinct influences of BOF slag dosage and Sasobit content on the static and dynamic mechanical properties of both HMRA and WMRA mixtures. Notably, significant effects are observed on static mechanical strength parameters (Marshall modulus and indirect tensile strength), attributable to both Sasobit warm-mixed modifier and BOF slag. Particularly noteworthy is their impact on Marshall modulus, where variations in both components significantly affect the mixtures’ resistance to high-temperature deformation. However, Sasobit warm-mixed modifier exerts a more pronounced effect on indirect tensile strength compared to BOF slag, suggesting its superior contribution to enhancing the mixture’s resistance against crack deformation.

The Sasobit warm-mixed modifier and BOF slag exhibit notable effects on the dynamic mechanical properties of rubber asphalt mixtures, specifically impacting elasticity modulus at 25°C, with corresponding p-values of 0.002 and 0.001, which is pivotal for enhancing the medium-temperature fatigue resistance of rubber asphalt mixtures. Both the Sasobit, BOF slag, and their interaction significantly affect the flow number and elasticity modulus at 40°C, suggesting a substantial impact on the elastic deformation behavior of WMRA mixtures. Notably, the dosage of BOF slag demonstrates highly significant effects (P<0.001), and the combined use of the Sasobit modifier and BOF slag synergistically enhances the high-temperature deformation resistance of WMRA mixtures. Conversely, at 50°C, only BOF slag exhibits a notable effect on the flow number (P<0.003). This phenomenon is likely due to the gradual dissolution of crystalline Sasobit wax at 50°C, leading to reduced high-temperature deformation resistance of warm-mixed rubber asphalt. Nonetheless, under high-temperature dynamic loading conditions at 50°C, BOF slag demonstrates the most substantial improvement in the resistance of rubber asphalt mixtures to permanent deformation.

**Fig 9:**
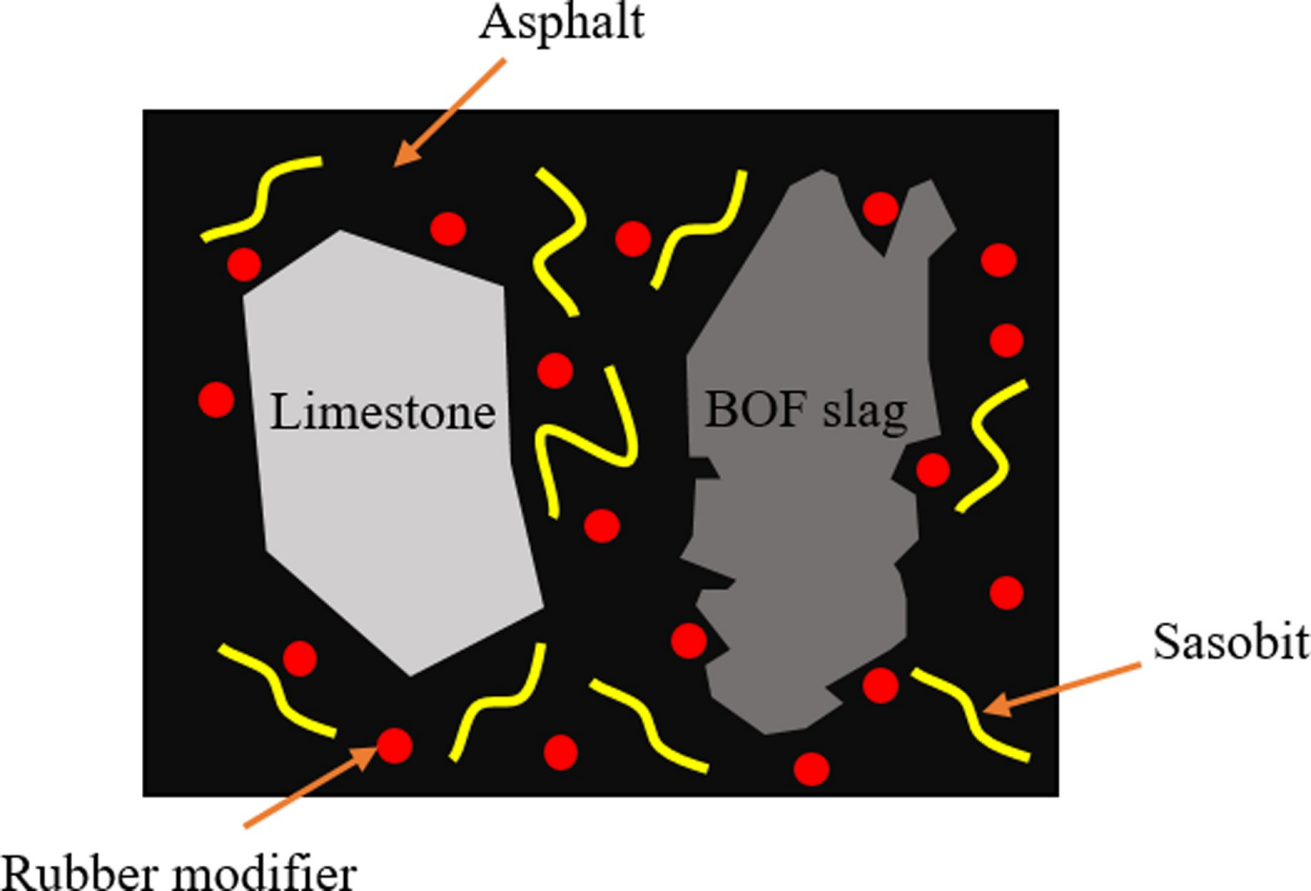


Therefore, the action mechanism of BOF slag and Sasobit modifier on rubber asphalt mixtures is illustrated in Fig 9. Compared to limestone aggregates, the rough surface texture of BOF slag enhances the contact force between the aggregates and rubber asphalt. Swelling rubber particles tend to adsorb into and fill the pores of BOF slag, thereby increasing the resistance to dynamic loads between the aggregates and asphalt. Additionally, the long-chain Sasobit modifier strengthens the cohesion between rubber particles and the asphalt matrix, and enhances the adhesion between BOF slag and rubber asphalt. The BOF slag and Sasobit modifier synergistically boost the resistance to deformation and fatigue of rubber asphalt, contributing to an extension in the lifespan of asphalt pavements.

## 5 Conclusion

1. BOF slag possesses excellent engineering aggregate characteristics, enabling its substitution for a large portion of coarse aggregates in both HMRA and WMRA mixtures. With increasing dosage of BOF slag, the resistance to permanent deformation of rubber asphalt mixtures under static and dynamic loads can be enhanced, and the optimal asphalt content of HMRA and WMRA is also increasing, which will substantially raise the cost of asphalt used.
2. Through static mechanical tests, BOF slag and Sasobit warm-mixed modifier enhance the Marshall modulus and indirect tensile strength of rubber asphalt mixtures at 60°C and 25°C, respectively, thereby improving the stiffness, resistance to deformation, and cracking resistance of the mixtures. ANOVA results indicate significant effects of BOF slag characteristics and Sasobit wax crystalline modification, with a weaker interaction between the two factors.
3. Dynamic mechanical tests reveal significant enhancements by BOF slag and Sasobit warm-mixed modifier on the modulus of elasticity at 25°C and 40°C, as well as the flow numbers at 40°C and 50°C. The increase in these metrics is notably higher compared to static mechanical indicators, thereby enhancing the medium-temperature fatigue resistance and resistance to rutting deformation of rubber asphalt mixtures, consequently expanding the elastic deformation range of WMRA and HMRA mixtures under repeated loading.
4. BOF slag and Sasobit warm-mixed modifier exhibit a significant improvement in dynamic mechanical performance indicators (P<0.01), with a significant interaction observed for the flow number and modulus of elasticity at 40°C, resulting in a synergistic enhancement of the resistance of rubber asphalt mixtures to dynamic loading deformation. As temperature increases, the influence of Sasobit warm-mixed modifier diminishes, while the impact of BOF slag on the resistance of mixtures to deformation under dynamic loading remains significant.

BOF slag and waste rubber, serving as industrial by-products in the steel and automotive sectors respectively, see an annual increase of billions of tons globally. Utilizing BOF slag and waste rubber to produce hot and warm mix rubber asphalt mixtures for road pavements holds significant engineering and environmental importance. Moving forward, ongoing research and monitoring will be required to assess the toxicity leaching of BOF slag and waste rubber within asphalt mixtures.
